# Characterization of Small Rubber Particle Protein 1 promoter from guayule (*Parthenium argentatum*)

**DOI:** 10.1186/s13104-025-07448-0

**Published:** 2025-09-02

**Authors:** Grisel Ponciano, Niu Dong, Chen Dong, Kumiko Johnson, Tina Williams, Delilah F. Wood, Grace Chen

**Affiliations:** United States Department of Agriculture, Agricultural Research Service-Western Regional Research Center, 800 Buchanan Street, Albany, CA 94710 USA

**Keywords:** Guayule, Small Rubber Particle Protein, Promoter, Natural rubber

## Abstract

**Objective:**

Guayule (*Parthenium argentatum*) is a rubber producing plant. Genetic engineering of guayule to improve natural rubber content requires the use of promoters functional in stem tissues where most of guayule natural rubber is produced.

**Results:**

We isolated a promoter region from a gene coding the Small Rubber Particle Protein 1. Transgenic guayule lines expressing the Small Rubber Particle Protein 1 promoter fused to β-glucuronidase reporter gene were developed. The promoter, active in leaf, stem and root tissues drives significant levels of transgene expression, especially in the stem tissue. The isolated Small Rubber Particle Protein 1 promoter is a new molecular element in the toolbox available for guayule improvement through genetic engineering strategies.

**Supplementary Information:**

The online version contains supplementary material available at 10.1186/s13104-025-07448-0.

## Introduction

The perennial woody shrub guayule (*Parthenium argentatum*) is currently cultivated in the southwestern U.S. for domestic natural rubber (NR) production. Economic viability of the guayule crop depends largely on rubber content as well as use of co-products, such as resin and bagasse (the leftover biomass after guayule rubber extraction). One approach to increase NR content in guayule is through metabolic pathway engineering targeted to the stem tissues where rubber is predominantly synthesized and stored. Guayule also synthesizes NR in roots, however, the current production practice requires leaving the root intact to allow for re-growth and future harvests [1]. Leaves synthesize very little NR [2]. Use of functional promoters in stem tissue is desirable for genetic modification of rubber biosynthesis related genes in guayule.

In all rubber producing plants, NR is synthesized at the surface of a cytoplasmic organelle known as a rubber particle (RP) [3, 4]. Several proteins and enzymes, including the rubber transferase enzyme complex, associate with the RP to synthesize NR directly, or indirectly to maintain RP structural integrity [5, 6]. One common RP-associated protein is the Small Rubber Particle Protein (SRPP), present in RPs from different plant species including Hevea (*Hevea brasiliensis*), rubber dandelion (*Taraxacum kok-saghyz* and the related species *T. brevicorniculatum*), lettuce (*Latuca sativa*) and guayule [7–10]. While the function of SRPP in rubber biosynthesis remains unclear, research has shown that its downregulation can affect rubber quality and quantity in rubber dandelion [10, 11], but not in lettuce [12]. Additionally, a structural role in the maintenance of the RP has been proposed [5]. Interestingly, *Srpp* homologs have been identified in non-rubber producing plant species and the homologs play a role in stress response [13, 14]. In guayule, cold stress induces *Srpp* expression [15]. In all the above rubber producing plants, the *Srpp* family is composed of at least 3 members. Promoters of some of the members have been cloned and functionally characterized [16–18]. Three putative *Srpp* genes have been identified in a transcriptome assembly of the tetraploid hybrid AZ-3 guayule line [19]. Only one of guayule *Srpp* genes has been cloned (AF541942, herein referred to as *Srpp1*) [9].

In this study, we isolated a guayule *Srpp1* (*PaSrpp1*) promoter, made a translational fusion with the reporter gene β-glucuronidase (*Gus*) and developed transgenic plants for functional characterization. The *PaSrpp1* promoter effectively drives the *Gus* gene in stem, as well as in young leaves and roots. The promoter is a new molecular tool useful for genetic engineering of guayule.

## Results and discussion

Breeding guayule is extremely difficult because of the inherent complex facultative apomictic mode of reproduction characteristic of the preferred lines for industrial use. Apomixis is asexual formation of a seed from the maternal tissues without fertilization [20]. Genetic engineering is an alternative tool to increase rubber content in guayule. One key element for genetic engineering is the use of promoter sequences to effectively drive gene expression in the appropriate tissue.

**Fig. 1:**
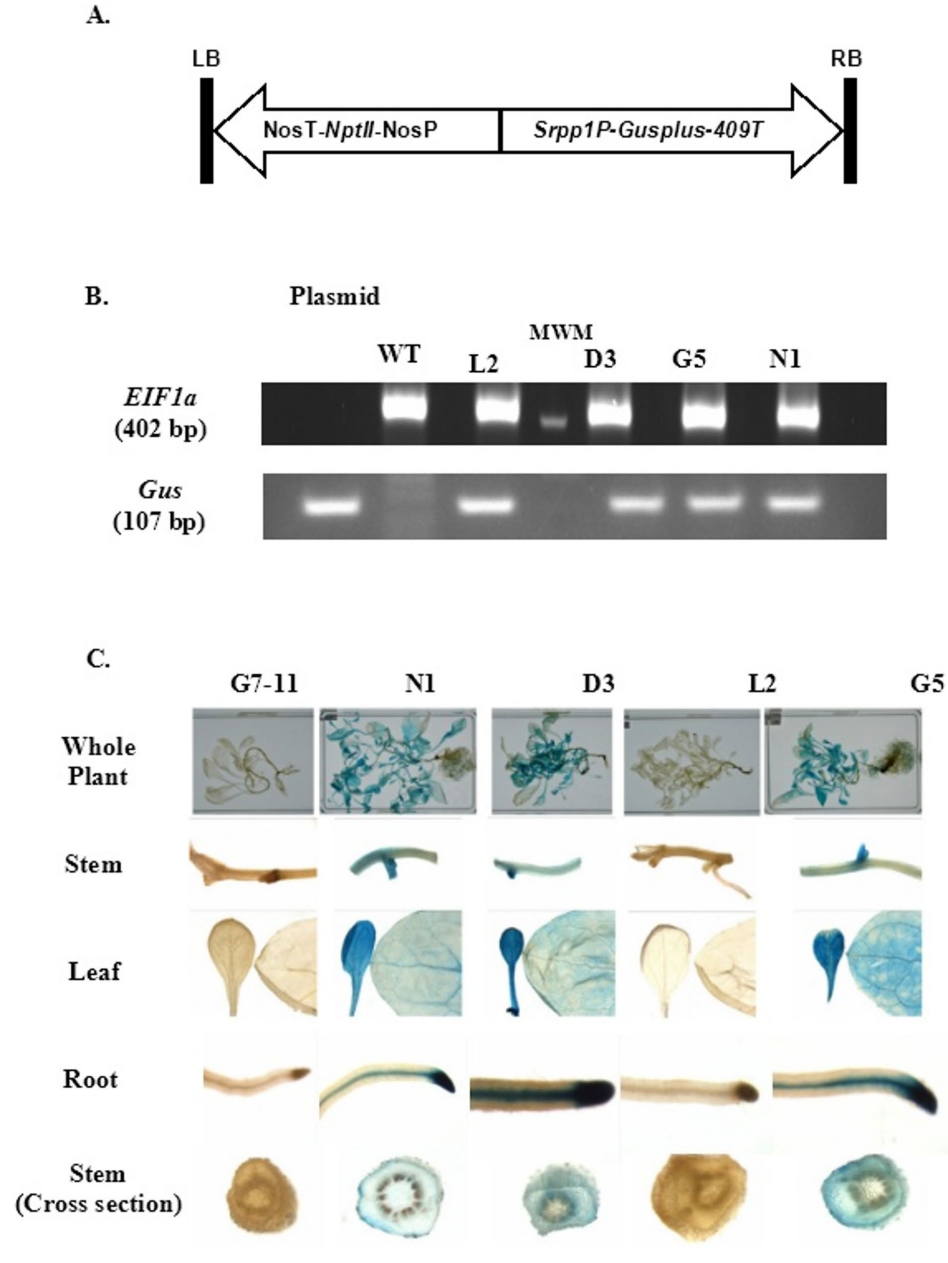
Transgenic guayule lines expressing *Gus* gene driven by *PaSrpp1* promoter. A, T-DNA region of *pND6::Srpp1P-Gusplus* plant transformation vector. SRPP1P, guayule *Srpp1* promoter; NptII, neomycin phosphotransferase II; *Gusplus*, β-glucuronidase; 409T, potato 409 terminator. B, Gel electrophoresis of gDNA PCR products confirm insertion of transgene. Transgenic lines: L2, D3, G5 and N1. WT, G7-11 untransformed guayule (negative control); plasmid, pND6::SrppP-Gusplus (positive control). Top panel, PCR products with endogenous control gene *EIF1a* (eukaryotic elongation factor 1a) primers. Bottom panel, PCR products with *Gus* primers. MWM, molecular weight markers (400 bp marker size on 2% agarose gel in top panel; 100 bp marker size on 4% agarose gel in bottom panel, too faint to show on photograph). C, GUS staining of 3-month-old control and transgenic guayule plants

**Fig. 2:**
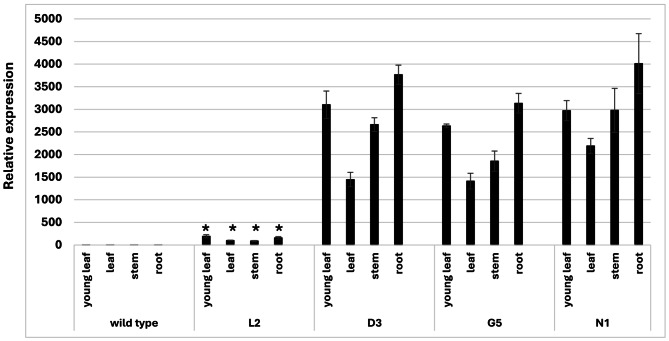
*Gus* relative gene expression in various organs of wild type and transgenic guayule lines. Plants were grown at constant 25 °C ambient temperature in growth chamber. Results are averages of three biological replicates with standard deviation as error bars. Wild type is non-transformed control line; L2, D3, G5, N1 are transgenic lines

We identified the promoter region of *PaSrpp1* in the genome of a diploid guayule line [21]. The putative *PaSrpp1* promoter (*PaSrpp1-P*) was cloned out and translationally fused with the *Gus* reporter gene (Fig. 1A) to generate stable transgenic guayule lines via *Agrobacterium*-mediated transformation. Four transgenic lines were generated and verified positive via genomic DNA PCR (Fig. 1B). All these lines were phenotypically similar to the non-transgenic wild type G7-11 control line. Activity of *PaSrpp1-P* was evaluated by GUS staining the entire plant (3-month-old) (Fig. 1C) and measuring *Gus* transcript levels in different tissues (Fig. 2). The reporter *Gus* transcript had high relative expression levels in three transgenic lines (D3, G5, and N1) while line L2 exhibited statistically significant lower expression levels in all organs (Fig. 2). The low expression in L2 correlates well with the very weak blue staining in all organs (Fig. 1C). Although line L2 is PCR positive, several factors affect transgene expression such as copy number, location of integration, complex regulatory mechanisms, and suppression by homolog sequences [22, 23]. The highest *Gus* expression in the transgenic lines was observed in roots, followed by young leaves, stems and mature leaves. GUS staining showed a strong expression in the vascular tissue of roots and root tips. *PaSrpp1-P* was more strongly expressed in young leaves than mature leaves (Figs. 1C and 2), indicating a role during leaf development. As the stem is the main organ for NR production in guayule, we specifically examined the cross section of stems. We found that *PaSrpp1-P* had strong activity in parenchyma tissues, including cortex, phloem and bark tissues (Fig. 1C). These tissues were demonstrated to be the sites for NR biosynthesis and storage [6]. The gene expression profiles in the stems make *PaSrpp1-P* useful for targeting NR biosynthesis in guayule stems.

**Fig. 3:**
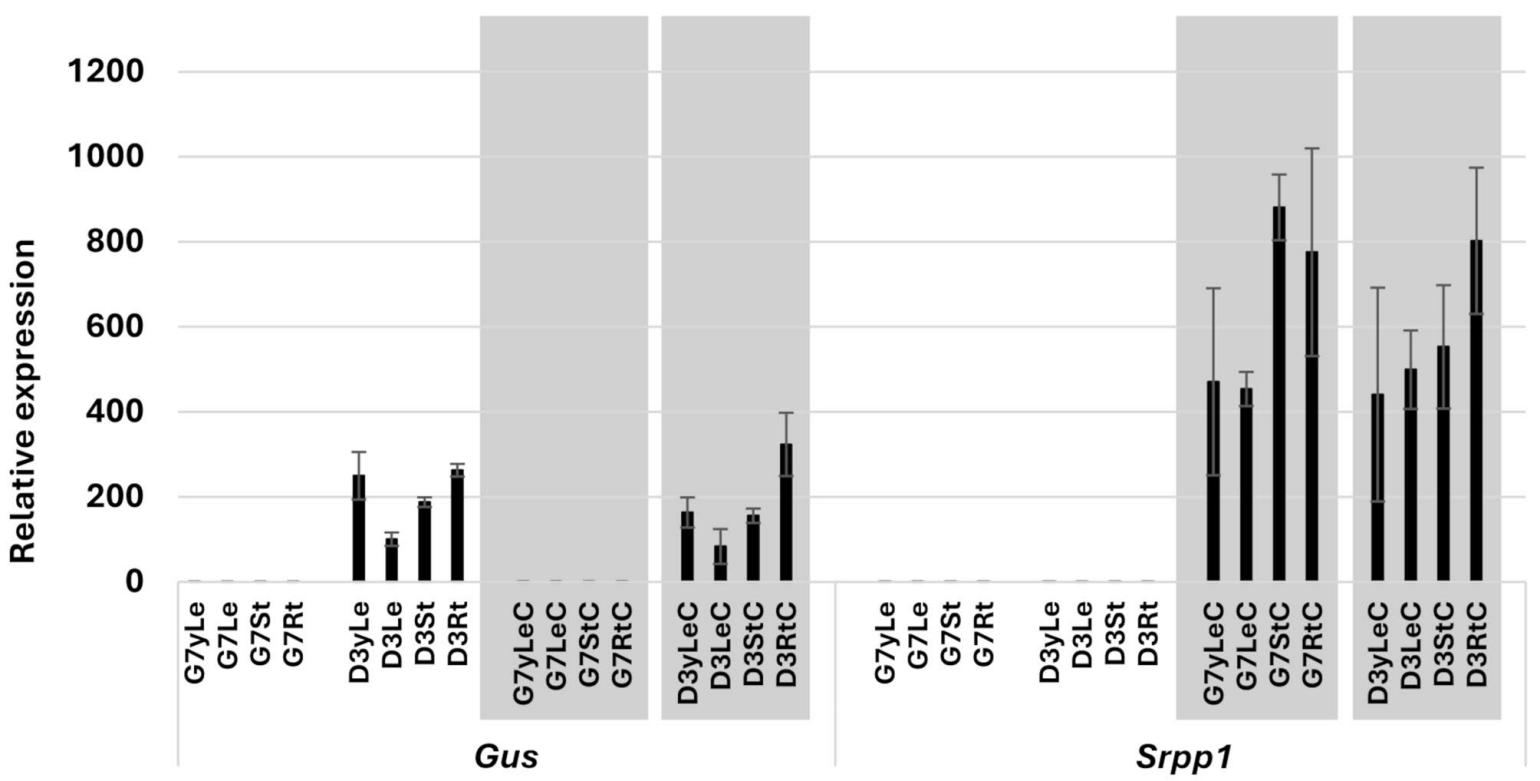
*Gus* and *Srpp1* relative gene expression in control (G7) and transgenic (D3) guayule lines. The 3-month-old plants were grown at 25 °C during daylight and 5 °C (grey area) during nighttime for five days. Bars are the average of three biological replicates with standard deviation as error bars. G7, G7-11 wild type; D3, transgenic line; yLe, young leaf; Le, leaf; St, stem; Rt, root; C, cold

The role of SRPP in plant stress response is well documented [13, 14, 24–26]. The promoter regions of both *TkSrpp* and *HbSrpp* have been shown to be regulated by light, wounding, hormone treatment and cold stress [16–18]. The in silico analysis (Supplementary Fig. S1) of the 1,740 bp *PaSrpp1* promoter region revealed the same *cis*-acting regulatory elements involved in hormone and light-responsiveness present in *Srpp* promoters from *HbSRPP* [17] and *TkSRPP* [18], but not stress-response motifs. A guayule transcriptome analysis identified several rubber biosynthesis transcripts up regulated by cold temperatures including *Srpp1* [15]. We were curious to know if the isolated *PaSrpp1* promoter is also responsive to cold temperatures. As shown in Fig. 3, no differences were observed for *Gus* transcript levels between cold and room-temperature (control) samples; however, guayule endogenous *Srpp1* transcripts were significantly higher in cold than controls. The results indicate that the cloned *PaSrpp1* promoter fragment does not confer cold responsiveness, and the observed induction of endogenous *Srpp1* expression is likely driven by additional *cis*-elements located outside the 1,740 kb promoter region. More stress-responsive motifs, including cold-responsive motifs, remain to be identified. Our results direct future experiments for identifying cold-responsive motifs and other *cis*-elements.

In conclusion, we isolated and demonstrated a *PaSrpp1-P* region that functions as a constitutive promoter. We also demonstrated that the native *PaSrpp1* promoter is cold-responsive. Our results are valuable in implementing genetic engineering approaches to improve NR content in guayule.

## Materials & methods

### Plant material and maintenance

Guayule (*Parthenium argentatum* A. Gray) G7-11 line (a selection from the AZ-2 guayule line) was the leaf source for *Agrobacterium*-mediated transformation. The seed (PI 599675) were obtained from the United States Department of Agriculture National Plant Germplasm System (www.ars-grin.gov). Transgenic and control lines were maintained in Magenta vessels in a growth chamber with a 16 h photoperiod (cool-white fluorescent lights ~ 50 µmol/m^2^/s), 23–27 °C, ambient relative humidity ~ 65%. Plants were propagated vegetatively every 2–3 months as previously described [27]. For cold treatment, 3-month-old transgenic lines and controls were moved to a temperature-controlled chamber, grown at 25 °C ambient temperature during daylight hours, and at 5 °C during nighttime for five days with a 12 h photoperiod.

### Transformation construct and transgenic guayule lines analysis

Guayule *Srpp1* promoter was identified in silico by homology search of diploid guayule genome with SRPP1 protein sequence (Accession No. AAQ11374) as query. The upstream region 5’ of *Srpp1* was used to design PCR primers 5’-CGGTTCTGTCAGTTCCAAAC-3’ and 5’- CATACCAATGCTAAGGAGGAC-3’ to clone out the putative promoter region of ~ 1,740 bp. Guayule G7-11 genomic DNA was the template for amplification with GoTaq^®^ Long PCR Master Mix Kit (Promega, Madison, WI, USA) following manufacturer instructions, and sequenced. The isolated promoter region sequence is available in GenBank, Accession PV754023. The promoter sequence was queried to PlantCARE [28] to determine whether it shares similarities with known cis-acting regulatory elements.

The plant transformation vector *pND6::Srpp1P-Gusplus* was constructed by removing the potato 409 promoter driving *Gusplus* gene from pND6 vector [29] and replacing it with guayule *Srpp1* promoter via *HindIII*-*XbaI* sites using primers 5’-AAGCTTCCCCCTTTTAATTATGATATG-3’ and 5’-GCGTCTAGAAGAACAGAAG-3’. Transgenic guayule lines were developed as previously described [25]. Insertion of T-DNA in transgenic lines were confirmed by genomic DNA polymerase chain reaction (PCR). REDExtract-N-Ampa Plant PCR kit (Sigma-Aldrich, Carlsbad, CA, USA) was used to extract genomic DNA and PCR. PCR primers specific to the *Gusplus* transgene (forward: 5’-CGAAGCGAGCAATGTGATGG-3’, reverse: 5’- GATCCGCAAGACGCATCAAC-3’) and guayule endogamous EF1a (forward: 5’-TCTCTTGGGCTCGTTGATCT-3, reverse: 5’-TCGAGGCTGGTATTTCCAAG-3’) were used to amplify the genomic DNA with predicted amplicon size of 107 bp for *Gusplus* and 402 bp for *EF1a*, respectively. The PCR reaction and product visualization (Supplementary Figs. S2 and S3) were performed as described previously [30].

### Quantitative PCR analysis

Total RNA from samples were extracted according to the instruction in kit Total RNA Isolation Kit (Ambion, Pittsburg, PA, United States). cDNA samples were prepared using QuantiTect Reverse Transcription Kit (QIAGEN, Valencia, CA, USA). Quantitative PRC (qPCR) reactions were performed as described previously [31]. PCR product specificity was confirmed by melting-curve analysis. Primer pairs for transgene *Gusplus* (forward: 5’-CGAAGCGAGCAATGTGATGG-3’, reverse: 5’- GATCCGCAAGACGCATCAAC-3’) and guayule internal reference gene *PaEF1a* (forward 5’CACAGCAAACCGACCAAGTG-3’; reverse, 5’CGACAGACGATCCGGTAAGG-3’) were tested with qPCR efficiency showing at 2.01 and 1.92, respectively. Relative gene expression levels were calculated as described [32].

### GUS staining and microscopy

Three month old guayule plants were gently removed from the Magenta vessels and immersed in a 50mL conical Falcon tube containing 30mL of GUS staining solution (0.1 M sodium phosphate buffer, pH 7.0; 0.5mM potassium ferrocyanide; 0.5mM potassium ferricyanide; 0.5% (v/v) Triton X-100; 0.15% (w/v) X-Gluc [5-Bromo-4-chloro-3-indoxyl-beta-D-glucuronide cyclohexylammonium salt]). Samples were stored overnight (> 18 h) at 37 °C in an incubator in the dark. The next morning, plants were destained with 95% ethanol solution in water with gentle shaking and ethanol solution changed every hour until the chlorophyll was completely removed. Plants were rinsed with water and stored in 10% glycerol solution in water until ready for photography and tissue collection for microscopy analysis. Whole plants were photographed with a high-resolution digital camera (Nikon-D7000). Stems, hand cut cross-sections of stems, leaves, and root tips were photographed using a Leica MZ16F stereo microscope (Leica Microsystems, Inc, Deer Park, IL, USA). Digital images were collected using a Micropublisher 6 digital camera (Teledyne Photometrics, Surrey, British Columbia, Canada) and Image-Pro Plus software (Media Cybernetics, Rockville, MD, USA).

### Limitations

Guayule transformation efficiency is extremely low and therefore a detailed dissection of *PaSrpp1* promoter fragments of different lengths to identify functional motifs is difficult. Further promoter testing will be best suitable on model plants such as Arabidopsis or tobacco.

## Supplementary Information

Below is the link to the electronic supplementary material.

Supplementary Material 1

Supplementary Material 2

Supplementary Material 3

## Data Availability

The promoter sequence associated with this work was submitted to GenBank, Accession PV754023, https://www.ncbi.nlm.nih.gov/nuccore/PV754023.

## Abbreviations

G7-11: Guayule allotetraploid line
*Gus*: β-glucuronidase reporter gene
NR: Natural rubber
RP: Rubber particle
SRPP: Small Rubber Particle Protein
