# Supplementary material for: Characterization of Small Rubber Particle Protein 1 promoter from guayule (Parthenium argentatum)
Source: BMC Res Notes. 2025 Sep 2;18:380. doi: 10.1186/s13104-025-07448-0 (PMC12406399; doi:10.1186/s13104-025-07448-0)
Supplement: Supplementary file 1 — Supplementary Material 1 [file 13104_2025_7448_MOESM1_ESM.pdf]

Fig. S1: Location of predicted *PaSrpp1* promoter *cis* elements using PlantCARE database.

```
+ CCCCCTTTTA ATTATGATAT GTTTTATAAT TAAATAATTT CTTATTTAAT TTAAACAATA AATCTTAAAA
- GGGGGAAAAT TAATACTATA CAAAATATTA ATTTATTAAA GAATAAATTA AATTTGTTAT TTAGAATTTT

+ AAAAATAAGT TTTATAAATG AAATATTTAT TGGAAATTGC GTTTTAAATA TGGTTTTGCA AATATATAAA
- TTTTTATTCA AAATATTTAC TTTATAAATA ACCTTTAACG CAAAATTTAT ACCAAAACGT TTATATATTT

+ TATTCACATG CAAACACGTA AATTAATAAA TTGAAATAAA AAAGAGAATG AATAATCATG GAATTTGATT
- ATAAGTGTAC GTTTGTGCAT TTAATTATTT AACTTTATTT TTTCTCTTAC TTATTAGTAC CTTAAACTAA

+ GATTAGTTGA AATTAAAATT ACAATAAATT TAAATAAAAA TACACATGAA GATCCATGAT ATTAGAATCT
- CTAATCAACT TTAATTTTAA TGTTATTTAA ATTTATTTTT ATGTGTACTT CTAGGTACTA TAATCTTAGA

+ CTCATACTTC ATAAAATCTC GTCCCGAAGG TTATTTAAGA ATAGGCTTCT GGAAAAAGAT ATGGGTATTT
- GAGTATGAAG TATTTTAGAG CAGGGCTTCC AATAAATTCT TATCCGAAGA CCTTTTTCTA TACCCATAAA

+ GGCTTTCATT TGGTCTTCAC GTTCCCAAGT GAATTATGGG CCATGTTTAG ATTCTCAACG AACTTTGACT
- CCGAAAGTAA ACCAGAAGTG CAAGGGTTCA CTTAATACCC GGTACAAATC TAAGAGTTGC TTGAAACTGA

+ ATTGGAATAC GACTTCGTTT GAGCATCTTG ACTTCCCGAT CCATAACCTC CACAAGTTTT TCTATGAAGT
- TAACCTTATG CTGAAGCAAA CTCGTAGAAC TGAAGGGCTA GGTATTGGAG GTGTTCAAAA AGATACTTCA

+ GCATTGAATC ATCAATATGT ATGTCTTCGA GCGAATGTGA AGGGTTTTCC CCTGCTAGGC ATTTATTGAG
- CGTAACTTAG TAGTTATACA TACAGAAGCT CGCTTACACT TCCCAAAAGG GGACGATCCG TAAATAACTC

+ GTTGGAAACA TGAAATGTTG GATGAAAATT GCTAAGCTCT TGAGGTAGAT CAAGCTTGTA TGCTTCCTTC
- CAACCTTTGT ACTTTACAAC CTACTTTTAA CGATTCGAGA ACTCCATCTA GTTCGAACAT ACGAAGGAAG

+ CAGATCCTTT TAAGGATATT AAAAGGACCT ACATAGCGTG GTGCTTGTTT CCCATTCTTT TCGAATCGAA
- GTCTAGGAAA ATTCCTATAA TTTTCCTGGA TGTATCGCAC CACGAACAAA GGGTAAGAAA AGCTTAGCTT

+ ACATCAATTT CAAAGGGGAT ACCTTAAGAA GTACGAGATC ACCGACTTGG AATTTCAAGG TTTGCGTCTT
- TGTAGTTAAA GTTTCCCCTA TGGAATTCTT CATGCTCTAG TGGCTGAACC TTAAAGTTCC AAACGCAGAA

+ TGGTCAAGGA GTTCCCCACC CATATGAATG TTTTTATTGA ATGTGCAGTA GACTCATATA TATATTTTAT
- ACCAGTTCCT CAAGGGGTGG GTATACTTAC AAAAATAACT TACACGTCAT CTGAGTATAT ATATAAAATA

+ TAAATCGCCC AAGACACATT CTAAAATGTA TATATCGTCT GCCATGTGTA TTGGAATTAC TAAAAATTCC
- ATTTAGCGGG TTCTGTGTAA GATTTTACAT ATATAGCAGA CGGTACACAT AACCTTAATG ATTTTTAAGG

+ AAAAATTGTT ATCACGTAAG CAGTAGATTA TAACTAAACC GTTCAATAAC TAAAATATGA TGGAATACTT
- TTTTTAACAA TAGTGCATTC GTCATCTAAT ATTGATTTGG CAAGTTATTG ATTTTATACT ACCTTATGAA

+ AAAAAAATTA AAAAAAATTA TAAAAAAGAG GGTGATTGGT TGAAGTTGAA ATAGGCCCCA CTTATTTCCC
- TTTTTTTAAT TTTTTTTAAT ATTTTTTCTC CCACTAACCA ACTTCAACTT TATCCGGGGT GAATAAAGGG

+ TTTGGCGCTC GTTAACATGT TGGCTGAGTA GCTAACTATG CCCTCATTGA TGGGATAAGT CGGTGTTCAG
- AAACCGCGAG CAATTGTACA ACCGACTCAT CGATTGATAC GGGAGTAACT ACCCTATTCA GCCACAAGTC

+ CGCTCCAATA CTGATTTTGG AGTAGCTAAC CATGCCCCTC CACGCCACCC ACAACACATC GGGTGCAGCG
- GCGAGGTTAT GACTAAAACC TCATCGATTG GTACGGGGAG GTGCGGTGGG TGTTGTGTAG CCCACGTCGC

+ TGGAGATCGG CGTGGGGCTA AAGGCCACGG TGAGGTGACG GGCTGTGATT GTGCCTTTGA ATCCTACCGT
- ACCTCTAGCC GCACCCCGAT TTCCGGTGCC ACTCCACTGC CCGACACTAA CACGGAAACT TAGGATGGCA

+ TTGTTAACGA CAGGGTTATT TAAAAAATAT ACGCTATGGT GGAGTCCACA CAACCACCGT CAACGTCCCC
- AACAATTGCT GTCCCAATAA ATTTTTTATA TGCGATACCA CCTCAGGTGT GTTGGTGGCA GTTGCAGGGG

+ ACCACACCGC GGATTTTTTG TCTTGGATTG AAATTCCACA TACGCTTAGG TGAAGGTCCA TGCTCCCATA
- TGGTGTGGCG CCTAAAAAAC AGAACCTAAC TTTAAGGTGT ATGCGAATCC ACTTCCAGGT ACGAGGGTAT

+ AGAACGTGAG CAAATAATAT GGTTTAAGCT AGACTTGCTT CTTAAGTATT TTGATTACTT GCTTTTGTCA
- TCTTGCACTC GTTTATTATA CCAAATTCGA TCTGAACGAA GAATTCATAA AACTAATGAA CGAAAACAGT

+ TATTCTCCAA GATCAAACTT TACCAAAATG ACATGCCACA TGTAATCATC ACAATAAGCG ATGAGAATTG
- ATAAGAGGTT CTAGTTTGAA ATGGTTTTAC TGTACGGTGT ACATTAGTAG TGTTATTCGC TACTCTTAAC

+ GGGGTTTTCA TTTTAAACCC TAAAGTCTTC TCGAATTTTC CGCAACAGAT CCGGGCCCTT GTATTAAAAA
- CCCCAAAAGT AAAATTTGGG ATTTCAGAAG AGCTTAAAAG GCGTTGTCTA GGCCCGGGAA CATAATTTTT

+ GCCAATATCA TTCCATTAGT TCCCAGCACC CTTCAATCTC TTCCTACTTT CTTCTCCTAT TCCTTATTGT
- CGGTTATAGT AAGGTAATCA AGGGTCGTGG GAAGTTAGAG AAGGATGAAA GAAGAGGATA AGGAATAACA

+ AATCTCCGTC ACTACAAACT TCTGTTCTTC TAGACGCT
- TTAGAGGCAG TGATGTTTGA AGACAAGAAG ATCTGCGA
```

KEY:

Motifs Found - Known associated function in Arabidopsis

- ABRE - Abscisic acid responsiveness
- ABRE3a
- ABRE4
- ARE
- AT~TATA-box
- AuxRR-core - Auxin responsiveness
- Box 4
- CAAT-box - Promoter enhancer
- CGTCA-motif - Methyl Jasmonate responsiveness
- ERE
- G-Box - Light responsiveness
- G-box - Light responsiveness
- GARE-motif - Gibberellin responsiveness
- HD-Zip 3 - Protein binding site
- MYB
- MYB-like sequence
- MYC
- Myb-binding site
- STRE
- TATA-box - Core promoter element of transcription
- TATC-box - Gibberellin responsiveness
- TCA-element - Salicylic acid responsiveness
- TCT-motif - Part of light responsiveness
- TGA-element - Auxin responsiveness
- TGACG-motif - Methyl Jasmonate responsiveness
- Unnamed__1
- Unnamed__4
- W box
- as-1
