# Supplementary material for: Characterization of Small Rubber Particle Protein 1 promoter from guayule (Parthenium argentatum)
Source: BMC Res Notes. 2025 Sep 2;18:380. doi: 10.1186/s13104-025-07448-0 (PMC12406399; doi:10.1186/s13104-025-07448-0)
Supplement: Supplementary file 2 — Supplementary Material 2 [file 13104_2025_7448_MOESM2_ESM.pdf]

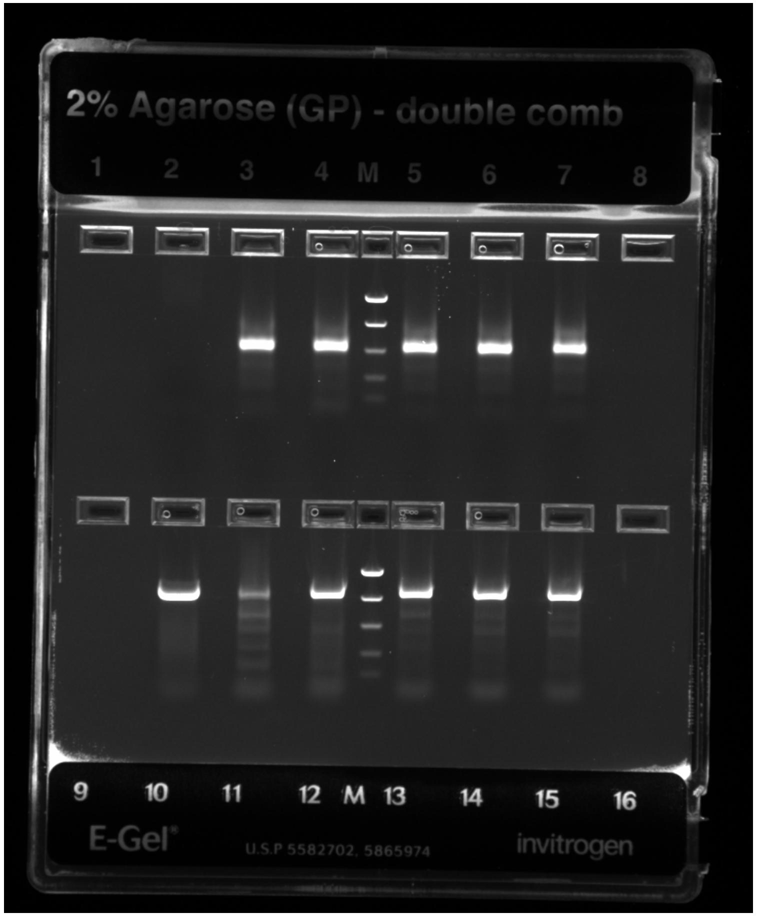

Gel well	Sample ID
1	Water
2	Agrobacterium (negative control)
3	Wild Type G7-11 (positive control)
4	Transgenic line L2
M	Molecular weight markers
5	Transgenic line D3
6	Transgenic line G5
7	Transgenic line N1
8	Water

Fig. S2: Full-length, unprocessed gel image of *EF1a* (Top gel) gDNA PCR products separated on a 2% agarose gel. PCR product size is 402 bp.

The bottom gel corresponds to a different PCR unrelated to the work reported here.
