# Supplementary material for: Characterization of Small Rubber Particle Protein 1 promoter from guayule (Parthenium argentatum)
Source: BMC Res Notes. 2025 Sep 2;18:380. doi: 10.1186/s13104-025-07448-0 (PMC12406399; doi:10.1186/s13104-025-07448-0)
Supplement: Supplementary file 3 — Supplementary Material 3 [file 13104_2025_7448_MOESM3_ESM.pdf]

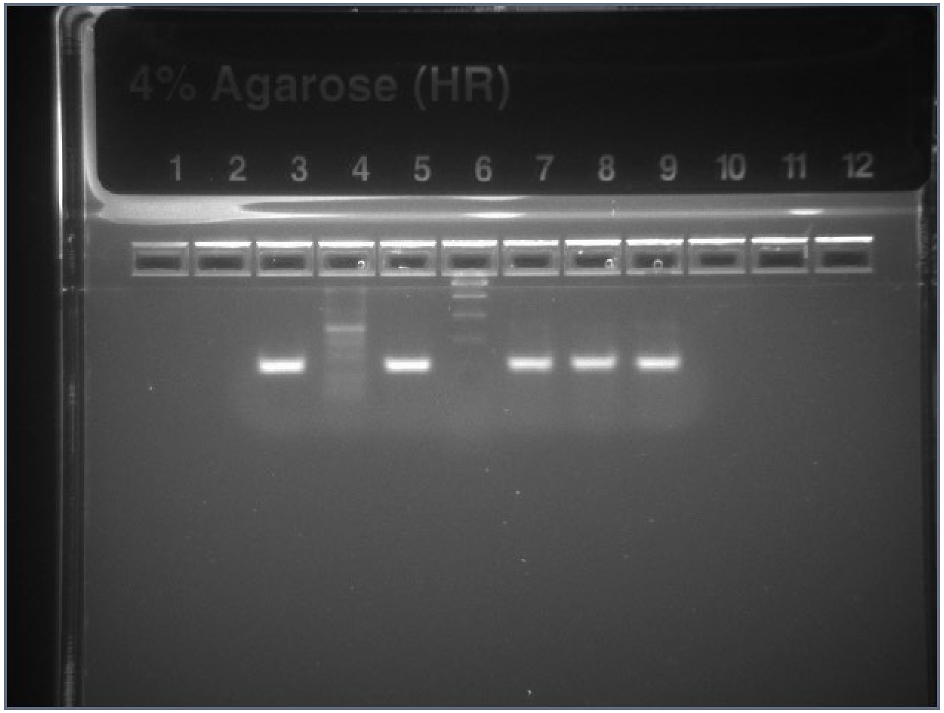

Gel well	Sample ID
1	Water
2	Water
3	Agrobacterium (positive control)
4	Wild Type G7-11 (negative control)
5	Transgenic line L2
6	Molecular weight markers
7	Transgenic line D3
8	Transgenic line G5
9	Transgenic line N1
10	Water
11	Water
12	Water

Fig. S3: Full-length, unprocessed gel image of *Gus* gDNA PCR products separated on a 4% agarose gel. PCR product size is 107 bp.
